# The thrombin receptor (PAR1) is associated with microtubules, mitosis and process formation in glioma cells^☆^

**DOI:** 10.1016/j.heliyon.2024.e33329

**Published:** 2024-06-19

**Authors:** Valery Golderman, Shany Guly Gofrit, Yanina Ivashko-Pachima, Illana Gozes, Joab Chapman, Efrat Shavit-Stein

**Affiliations:** a Department of Neurology, The Chaim Sheba Medical Center, Ramat Gan, 52621, Israel; b Department of Neurology and Neurosurgery, Faculty of Medical and Health Sciences, Tel Aviv University, Tel-Aviv, 6997801, Israel; c Elton Laboratory for Molecular Neuroendocrinology, Department of Human Molecular Genetics and Biochemistry, Faculty of Medical and Health Sciences, Adams Super Center for Brain Studies and Sagol School of Neuroscience, Tel Aviv University, Tel Aviv, 6997801, Israel; d Robert and Martha Harden Chair in Mental and Neurological Diseases, Faculty of Medical and Health Sciences, Tel Aviv University, 6997801, Israel; e The TELEM Rubin Excellence in Biomedical Research Program, The Chaim Sheba Medical Center, Ramat Gan, 52621, Israel

**Keywords:** Thrombin, PAR1, Microtubule, Glioblastoma multiforme, Mitosis

## Abstract

The cell surface protease-activated receptor 1 (PAR1) is overexpressed in glioblastoma multiforme (GBM). We studied the function and structure of intracellular microtubule (MT) and PAR1 in a tubulin-mediated process. We found that exposure to thrombin increased the percentage of proliferative, S, and M phases cells, affected morphology, and increased process elongation. PAR1 antagonist inversely affects these measures, increases tubulin end-binding protein 3 (EB3) mRNA expression in C6 cells, and reduces EB3 comet length, track length, and duration in neuroblastoma cells. In addition, immunofluorescence staining suggests that PAR1 is in close association with the MT α-tubulin and with coagulation cascade proteins during cell division stages. Our findings support PAR1 involvement in MT dynamics.

## 1 Introduction

Hypercoagulability is a common feature of malignancy, as evidenced by a 10–15 % prevalence of thromboembolism in cancer patients [1]. Production of microparticles containing high amounts of tissue factor (TF) by tumor cells contributes to thrombin generation and hypercoagulation [2]. Malignant cells produce inflammatory cytokines and attract inflammatory cells [3], promoting hypercoagulation due to the vast interaction with the coagulation systems [4]. Neutrophils activation by tumor cells leads to the creation of neutrophil extracellular traps (nets) which promote thrombin generation and hypercoagulability alongside tumor-cell migration, invasion, and angiogenesis [5]. The hypercoagulation state induced by the tumor supports its progression [6]. Activation of the thrombin G protein-coupled receptor (GPCR), protease-activated receptor 1 (PAR1) has been found to promote proliferation and invasion in gastric malignant cells [7] and signaling of the NFκB pathway as well as the production of IL-8 and IL-6 in prostate malignant cells [8]. The plethora of evidence has led to the exploration of anti-coagulation therapies as a possible anti-malignancy treatment.

Glioblastoma multiforme (GBM) is an aggressive tumor with a poor prognosis [9] presenting an unmet challenge. PAR1 is physiologically and intrinsically expressed in the central nervous system (CNS) mostly on glia cells and is located on astrocytic end-feet at the synapse [10]. Activation of PAR1 results in the creation of cell-free areas in hippocampal culture due to its effect on astrocyte morphology [11] and affects astrocytic neuropil in glutamatergic synapses resulting in changes in synaptic transmission [12]. PAR1 is present and overexpressed in GBM tumors and glioma cells [13,14], and its activation promotes tumor cell proliferation [15]. Thrombin/PAR1 pathway inhibition has been previously reported by our group to reduce thrombin activity and proliferation of glioma tumor cells *in-vitro* and to reduce edema volume with improved survival *in-vivo* [16]. The evidence thus supports PAR1 pathway involvement in glial morphology changes and increased proliferation leading to malignancy progression and edema expansion and suggests this pathway as a target for intervention in the treatment of GBM [[16], [17], [18]].

Microtubules (MTs) are protein polymers that are part of the cellular cytoskeleton. MTs are involved in various cellular events such as mitosis, migration, and process formation [19]. Many malignancy-associated cellular activities are MTs mediated, such as the post-translational changes in MTs that are known to promote tumor growth, angiogenesis, cell migration, and invasion [19].

From a mechanistic point of view, it is important to understand whether the thrombin/PAR1 pathway effects on the cellular morphological and proliferative process are in correlation to MTs dynamics. Previous data indicates several membrane bound receptors interact and affect the MT trafficking system, including tyrosine kinase receptors such as endothelial growth factor receptor (EGFR) [20], and the β2 adrenergic GPCR [21]. The PARs are known to be mainly localized at the cell membrane and internalized in vesicles following activation [22]. Previous data demonstrated that PAR2, a member of the PARs family, and a known plasma membrane protein, can relocate to the nucleus via microtubule depended shuttle, to exert a nuclear effect [23].

In the present study, we examined the effect of coagulation proteins on MTs dynamics in glioma cells. We demonstrate novel findings linking the coagulation pathway proteins and PAR1 to glioma cell mitosis and MT processes changes. Functionally, PAR1 modulation affects glioma cell morphology, proliferation, and migration through MT end-binding protein 3 (EB3) expression and MTs dynamics. During glioma cells’ mitosis, PAR1 is closely associated with α-tubulin, in contrast to it being mainly on the cell membrane in non-proliferating cells. In addition, coagulation factors, both from the intrinsic and the extrinsic pathway, as well as activated protein C (aPC) and endothelial protein C receptor (EPCR) are present in a similar association with the MT of the mitotic cells. These results shed new light on the role of the PAR1 pathway in cell cycle and MTs dynamics and support its modulation as a pharmacological target in malignancy treatments.

## 2 Results

### 2.1 Changes in cell proliferation following PAR1 modulation

**Fig. 1:**
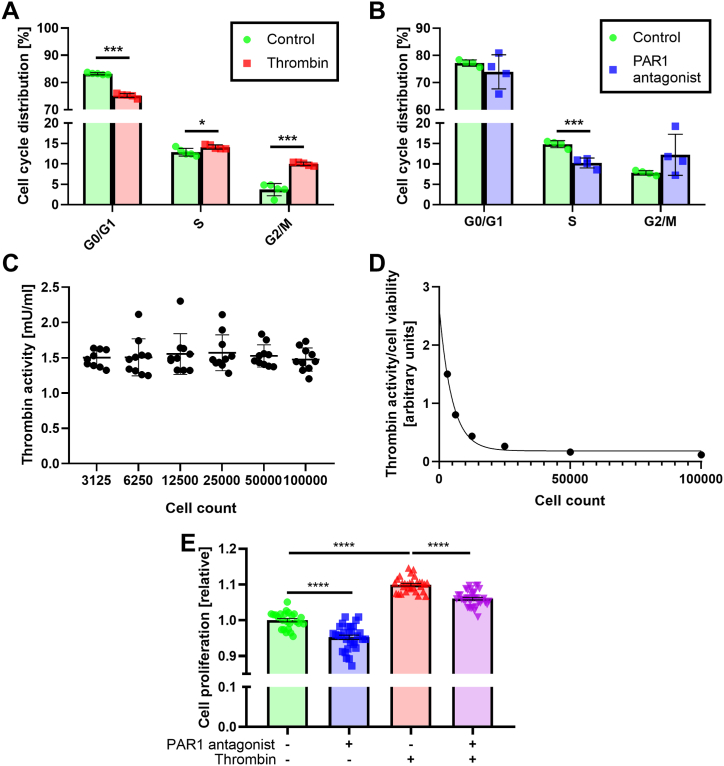
The effect of PAR1 modulation on cell-cycle and cell proliferation in C6 cells: A. Quantification of distribution in the different phases of the cell cycle in control and thrombin (1U/ml) treated C6 cells, N = 5, *t*-test. B. Quantification of distribution in the different phases of the cell cycle in control and PAR1 antagonist (100 nM) treated C6 cells. N = 5, *t*-test. C. Thrombin activity in the medium in various cell densities. N = 10, Kruskal-Wallis test. D. Thrombin activity per cell in various cell densities. E. Cell proliferation following combinations of thrombin (1U/ml) and PAR1 antagonist (100 nM) treatments. N = 24, 30 and 36, one-way ANOVA with Tukey's post-hoc analysis. Results are presented as mean ± SEM. *p < 0.03, ***p < 0.0008, ****p < 0.0003.

PAR1 was previously linked to tumor cell proliferation, and proliferation is an MT driven process. We first assessed the effect of PAR1 modulation on mitosis. Thrombin significantly modulated the differential distribution (proportion) of the cells between the cell-cycle stages. It decreased the relative portion of cells at the G0/G1 phase (75.18 ± 0.33 % and 83.21 ± 0.16 %, respectively, p < 0.0008), increased the relative parts in S phase (14.11 ± 0.23 % and 12.85 ± 0.41, respectively, p < 0.03) and G2/M phase, compared to control (9.998 ± 0.196 % and 3.68 ± 0.67 %, respectively, p < 0.0008, Fig. 1A). In contrast, PAR1 antagonist treatment caused a significant decrease in the relative part of cells at S phase, compared to control (10.22 ± 0.61 % and 14.82 ± 0.43 %, respectively, p < 0.0008 Fig. 1B).

As was described previously, the proliferation rate of C6 cell line decreases when cell density is increased [24]. Therefore, we studied whether this proliferation-density correlation is associated with thrombin activity. As can be seen in Fig. 1C, C6 cells retain a constant thrombin activity regardless of their density. Accordingly, normalizing this thrombin activity to cell-density emphasizes that individual cells secrete higher levels of thrombin activity at low cell-density (Fig. 1D). We further examined this point by measuring cell proliferation following PAR1 modulation. As shown in Fig. 1E, PAR1 antagonist treatment caused significantly decreased proliferation (0.95 ± 0.006 and 1 ± 0.005, respectively, p < 0.0003), and thrombin treatment caused significantly increased proliferation (1.1 ± 0.005 and 1 ± 0.005, respectively, p < 0.0003), compared to controls. When thrombin and PAR1 antagonist were applied together, cell proliferation increase was significantly prevented compared to thrombin alone (1.06 ± 0.004 and 1.1 ± 0.005, respectively, p < 0.0003), but remained significantly increased compared to controls (1.06 ± 0.004 and 1 ± 0.005, respectively, p < 0.0003).

### 2.2 Morphological changes following PAR1 modulation

**Fig. 2:**
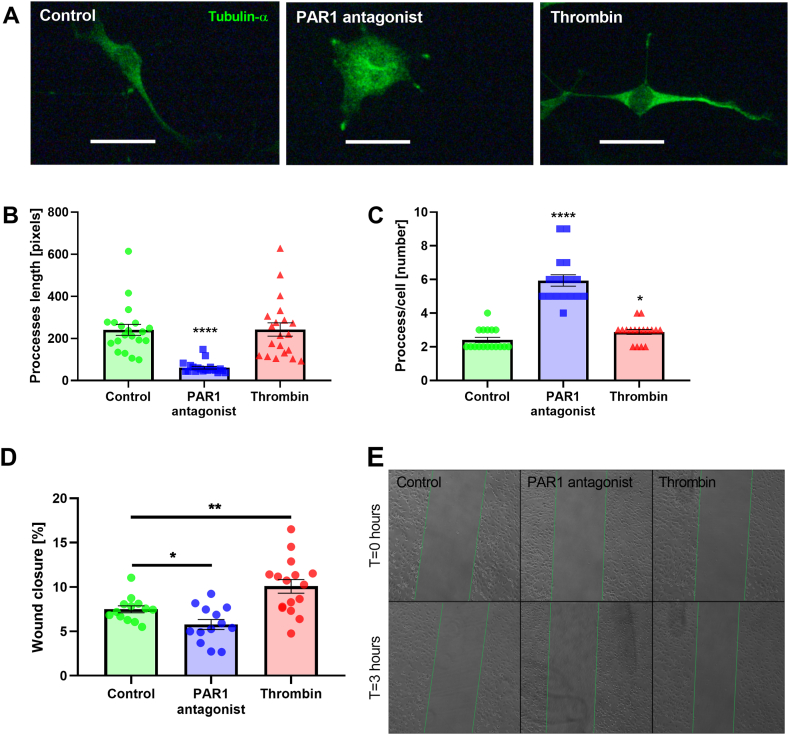
PAR1 modulation effect on process and cell migration: A. Representative α-tubulin (green) staining of C6 control, PAR1 antagonist (100 nM) and thrombin (1U/ml) treated cells. Scale bar – 10 μm. B. Process length in control, PAR1 antagonist and thrombin treated C6 cells. N = 20, Kruskal-Wallis test with Dunn's post-hoc analysis. C. Number of process per cell control, PAR1 antagonist and thrombin treated C6 cells. N = 17, Mann-Whitney test. D. Percent of wound closure following PAR1 antagonist (100 nM) and thrombin (1U/ml) treatments. N = 13 and 16. *t*-test. E. Representative images of wound closure in untreated, PAR1 antagonist and thrombin treated cells at t = 0 h (upper panel) and t = 3 h (lower panel). Results are presented as mean ± SEM. *p = 0.03, **p = 0.0094, ****p < 0.0001. (For interpretation of the references to colour in this figure legend, the reader is referred to the Web version of this article.)

Analogous to their role in cell division, MTs play a crucial role in process formation and cell migration. We therefore assessed the effect of PAR1 modulation on C6 cell line processes. We applied thrombin and PAR1 antagonist for 24 h and then visualized the α-tubulin positive processes. As can be seen in Fig. 2, the PAR1 antagonist caused a significant decrease in α-tubulin positive process length (61.25 ± 6.2 pixels and 240.8 ± 26.1 pixels, respectively, p < 0.0001, Fig. 2A,B) and a significant increase in the number of processes per cell, compared to control (5.94 ± 0.34 and 2.41 ± 0.15, respectively, p < 0.0001, Fig. 2A–C). In contrast, thrombin did not affect the length of the processes but significantly increased the number of processes per cell, compared to control (2.88 ± 0.15 and 2.41 ± 0.15, respectively, p < 0.03, Fig. 2A–C).

We measured cell migration using the scratch assay. We found that the PAR1 antagonist significantly decreased the percent of wound closure compared to controls (5.77 ± 0.57 % and 7.5 ± 0.39 %, respectively, p < 0.02, Fig. 2D and E). In contrast, thrombin significantly increased the percent of wound closure compared to control (10.08 ± 0.77 % and 7.5 ± 0.39 %, respectively, p < 0.01, Fig. 2D and E).

### 2.3 mRNA expression of major coagulation proteins and MT end-binding proteins

**Fig. 3:**
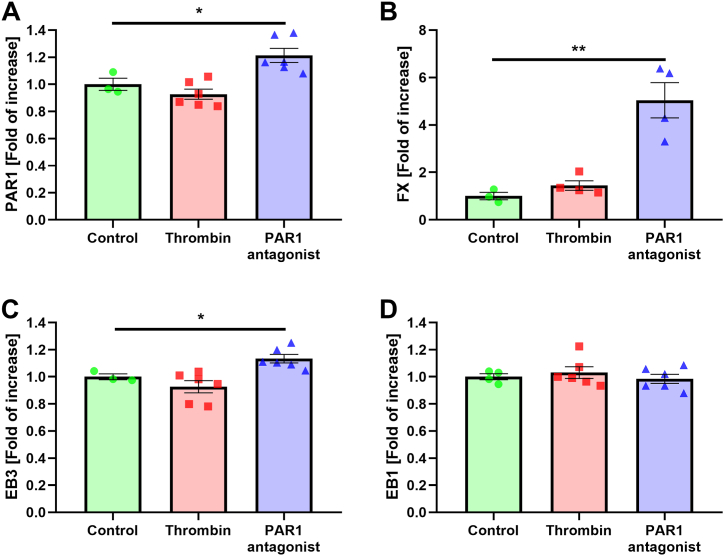
mRNA expression following PAR1 modulation: PAR1 (A), FX (B) EB3 (C) and EB1 (D) mRNA expression in control, PAR1 antagonist (100 nM) and thrombin (1U/ml) treated C6 cells. N = 3 to 7. Results are presented as mean ± SEM. Using one-way ANOVA with post-hoc analysis. Results for EB3 were analyzed using *t*-test. *p < 0.035, **p = 0.006.

The dynamics of MTs are regulated by MT end-binding (EBs) proteins. We therefore, evaluated whether PAR1 modulation affects the intrinsic mRNA expression of EBs proteins, and major coagulation proteins in C6 cells. Interestingly, thrombin (1 U/ml, for 3 h) did not significantly change the mRNA expression of PAR1, FX, EB3 and EB1 (Fig. 3A–D). In contrast, PAR1 antagonist (100 nM, for 3 h) significantly increased mRNA expression of PAR1 (1.2 ± 0.05 and 1 ± 0.05, respectively, p < 0.03, Fig. 3A), FX (5.04 ± 0.74 and 1 ± 0.16, respectively, p < 0.006, Fig. 3B) and EB3 (1.13 ± 0.03 and 1 ± 0.02, respectively, p < 0.027, Fig. 3C) compared to controls.

### 2.4 PAR1 antagonist affects tubulin dynamics

**Fig. 4:**
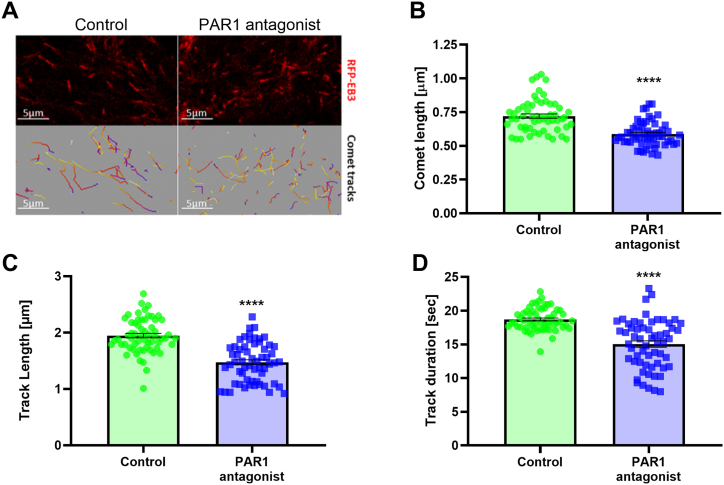
The effect of PAR1 antagonist on comet length, track length and duration. A. Representative live imaging of N1E-115 cells transfected with RFP-EB3 (red) in control and PAR1 antagonist (100 nM) treated cells. Quantification of comet length (B), track length (C) and truck duration (D) in control and PAR1 antagonist (100 nM) treated cells. N = 57 to 60, *t*-test. Results are presented as mean ± SEM. *p < 0.035, **p = 0.006. (For interpretation of the references to colour in this figure legend, the reader is referred to the Web version of this article.)

Since the PAR1 antagonist had a selective effect on EB3 expression, we evaluated the effect of the PAR1 antagonist on the microtubular cytoskeleton by performing time-lapse imaging of individual MTs in living cells. We used a well-established method for measuring MTs dynamics in neuroblastoma cells by measuring the mobility of RFP-tagged EB3 that binds to MT plus-ends [25] (Fig. 4A). The parameters that were assessed are EB3 comet-like structures length, growth track length and duration. These parameters reflect the length of MT growing tips, and the lengths and duration of the MT growing events, respectively. Before performing this assay, we conducted immune stain of the cells for PAR1 and confirmed a similar staining pattern during mitosis and to those found in C6 cells. The PAR1 antagonist significantly decreased EB3 comet length (0.59 ± 0.011 μm and 0.72 ± 0.016 μm, respectively, p < 0.0001, Fig. 4B), track length (1.47 ± 0.045 μm and 1.95 ± 0.039 μm, respectively, p < 0.0001, Fig. 4C), and track duration, compared to control (15.04 ± 0.47 s and 18.58 ± 0.24 s, respectively, p < 0.0001, Fig. 4D).

### 2.5 Co-localization of PAR1 and α tubulin during mitosis

**Fig. 5:**
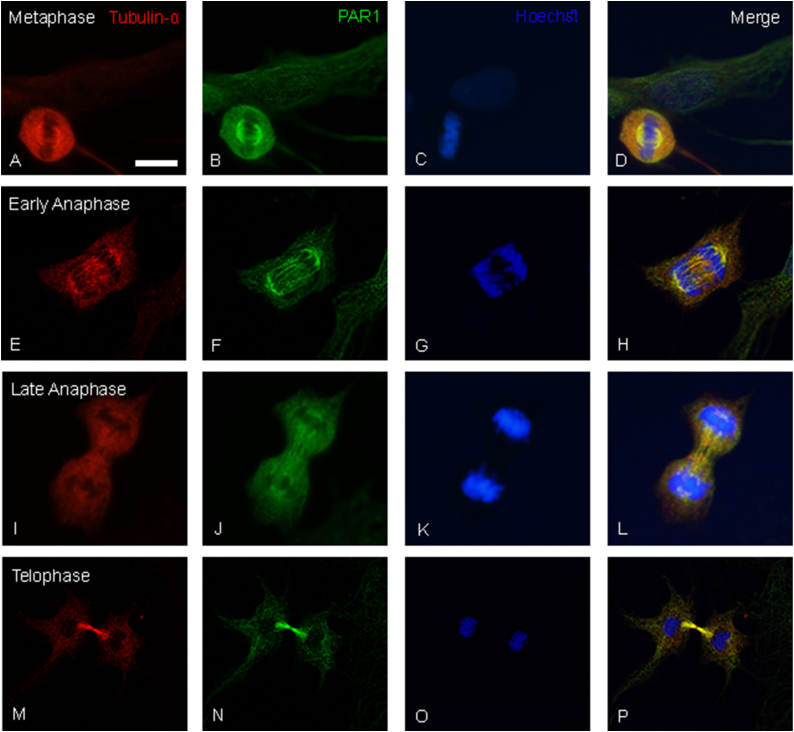
PAR1-tubulin α co-localization: Representative images of C6 cells stained to α-tubulin (red), PAR1 (green) and Hoechst (blue) in different stages of mitosis. A-D. Metaphase, r = 0.953. E-H. Early anaphase, r = 0.867. I-L. Late anaphase, r = 0.953. M-P. Telophase, r = 0.864. Scale bar – 10 μm. (For interpretation of the references to colour in this figure legend, the reader is referred to the Web version of this article.)

Since our results demonstrated a link between PAR1 modulation and MT dynamics, we examined the location of PAR1 and coagulation factors in C6 cells. Interestingly, co-staining of α-tubulin and PAR1 revealed a significant and differential co-localization of these proteins during all mitosis stages (Fig. 5).

Fig. 5 shows staining for Tubulin α (5A, E, I, M), PAR1 (5B, F, J, N), Hoechst (5C, G, K, O) and a merge of all channels (5D, H, L, P), during different stages of the cell devition. PAR1 is mostly detected in close association with the metaphase plate kinetochore MTs (Fig. 5B) and during early anaphase (Fig. 5F). During late anaphase (Fig. 5J) and telophase (Fig. 5N), PAR1 is detected near the bipolar MTs that assemble the central spindle. The co-localization of PAR1 and α-tubulin is presented in Fig. 5D–H, L, and P, indicated by yellow staining and further quantified (metaphase: r = 0.953, early anaphase: r = 0.867, late anaphase: r = 0.953, telophase: r = 0.864). It is important to note that this co-localization was detected using two different anti-PAR1 antibodies (Table S1), to ensure the specificity of the staining. Moreover, additional glioma cell lines presented a similar pattern of PAR1 staining, and co-localization with α-tubulin, including CNS1(rat glioma) and U87 (human glioma).

### 2.6 The presence of coagulation cascade proteins during the cell cycle

**Fig. 6:**
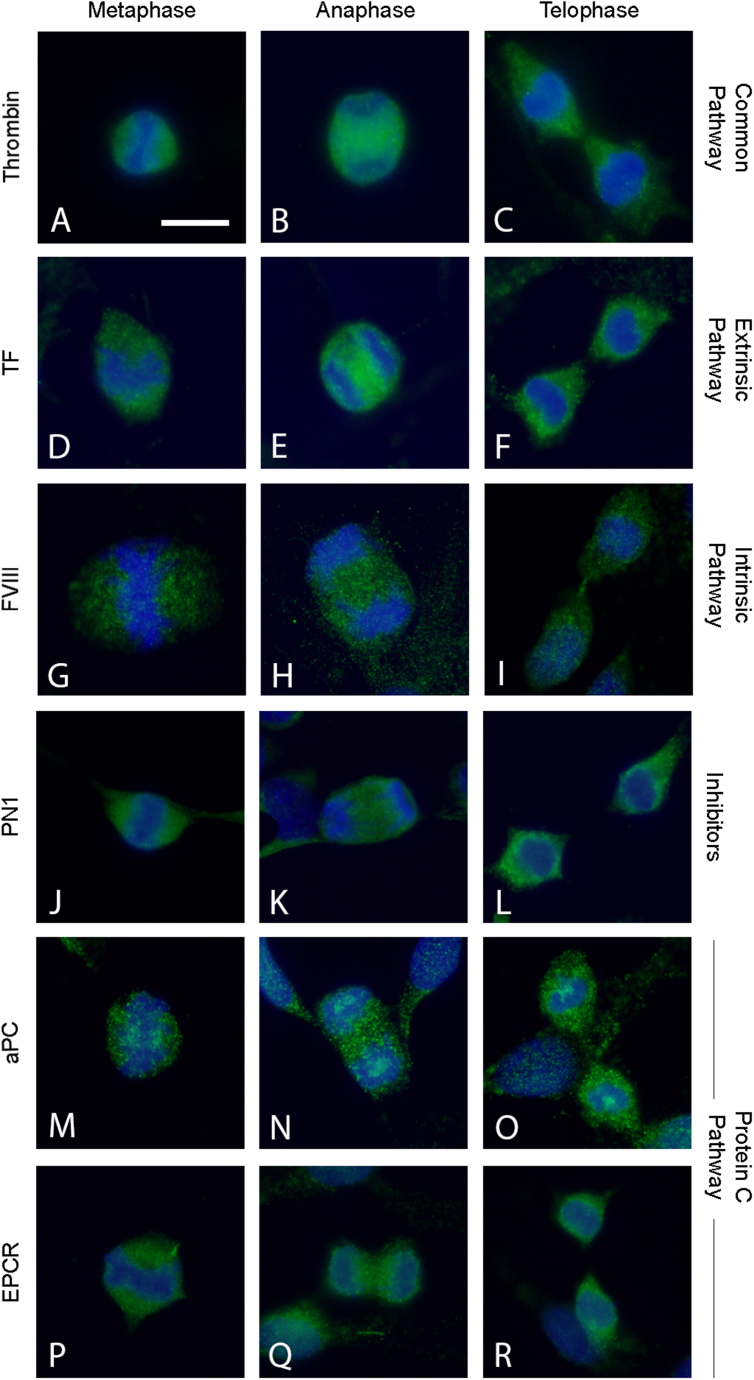
Coagulation factors in mitosis: Representative images of C6 cells stained to thrombin (A–C), tissue factor (TF, D-F), factor VIII (FVII, G-I), protease nexin-1 (PN-1, J-L), activated protein C (aPC, M − O), endothelial protein C receptor (EPCR, P–R) (all green) and Hoechst (blue) in different stages of mitosis. Scale bar – 10 μm. (For interpretation of the references to colour in this figure legend, the reader is referred to the Web version of this article.)

Following the co-localization of PAR1 and α-tubulin, we further evaluated other coagulation factors’ differential localization during the mitosis process stages. Both thrombin and its glial-derived inhibitor the protease nexin-1 (PN1) and factors from the intrinsic (FVIII) and the extrinsic pathway (TF) are present and elevated during all stages of the mitosis cell cycle (Fig. 6). In metaphase, thrombin, TF, and PN1 presented defuse staining all over the cell (Fig. 6. A, D, and J). In contrast, FVIII was mostly concentrated close to the metaphase plate, near the kinetochore MTs (Fig. 6G). In anaphase, strong staining of thrombin, TF, FVIII, and PN1 was found between the daughter chromatids (Fig. 6B–E, H, K). In telophase, TF and PN1 were largely absent at the central spindle area (Fig. 6F and L). Thrombin staining was absent only in the contractile ring area (Fig. 6C). Interestingly, FVIII staining was concentrated at the central spindle area, very similar to PAR1 (Fig. 6I). Although PAR1 is known to be activated by thrombin, it is also activated by several additional ligands [26] including activated protein C (aPC). Therefore, we evaluated the presence and specific localization of aPC and its receptor endothelial protein C receptor (EPCR) in C6 glioma cells during mitosis stages by immunofluorescence. As can be seen in Fig. 6, both aPC and EPCR stainings were detected during mitosis. Moreover, aPC is located close to the centrosome during all stages of mitosis (Fig. 6M − O) and absent from the central spindle area during the telophase (Fig. 6O). EPCR presented a very diffused staining pattern during all stages of mitosis and, like aPC, was absent from the central spindle area during the telophase (Fig. 6P-R).

## 3 Discussion

In this study we examined the function of PAR1 in MT dynamics and cell cycle progression during mitosis stages. We found that PAR1 activation increases the percentage of cells in division phases and PAR1 inhibition decreases the percentage of these phases. This effect was further supported by increased cell proliferation following thrombin treatment and decreased cell proliferation following PAR1 antagonist treatment. When thrombin was applied together with the PAR1 antagonist, the increase in cell proliferation was smaller. This observation suggests that PAR1 activation is crucial for cell proliferation, but it is possible that other PARs may be involved as well. In addition, we found that tumor cells at low densities secrete more thrombin per cell compared to cells at high densities. It is known that cells at low densities have a higher proliferation rate [24]. This data suggests that cells maintain optimal thrombin concentration to allow maximal proliferation. Whether these findings are directly linked to PAR1/MT interplay is a subject for future research.

In addition to cell division, MTs are responsible for cell migration and process dynamics. PAR1 was previously reported to be involved in cytoskeleton dynamics, via the Rho intracellular signaling pathway in endothelial cells [27], and in prostate malignant cells [28]. We therefore examined the effect of PAR1 activation and inhibition on process dynamics and cell migration. We found that PAR1 activation by thrombin significantly increases the number of processes per cell, without significantly affecting their length. In contrast, PAR1 inhibition significantly increases the number of processes, but also significantly decreases their length. In addition, PAR1 inhibition decreases cell migration, while PAR1 activation increases it. It is important to note that the PAR1 antagonist used in the present study is relatively lipid soluble and probably penetrates better into cells while thrombin is a much larger molecule and has a limited penetration capability, which may explain the lack of thrombin effect on process length. Higher levels of the PAR1 antagonist cause cell death in these cultures. Taken together, our data supports the involvement of thrombin-PAR1pathway in MT dynamics and therefore in process formation and cell migration. Like the findings regarding the proliferation, the question about the preceding complex chain of events leading to the PAR1 induced MT dynamics still remains.

We examined gene expression following PAR1 activation and inhibition. We found that PAR1 inhibition significantly increases FX and PAR1 mRNA expression, probably as a negative feedback loop. This data further supports the intrinsic endogenous production and regulation of coagulation factors and proteins in glioma cell lines. Interestingly, PAR1 inhibition also significantly increases the expression of EB3. The MT-associated protein (MAP) EB3 is an MT end-binding protein that is known to be abundant in the CNS. EB3 binds mostly to the growing plus ends of the MTs and regulated their dynamics, including in mitosis [29]. Changes in gene expression of MAP following PAR1 inhibition further support the hypothesis that PAR1 affects MTs through MAPs. This possibility was further supported by the effect of PAR1 inhibition on MTs dynamics demonstrated by tracking of EB3 comet-like structures on newly polymerized MT plus-ends. PAR1 inhibition decreased EB3-comet length, track length and duration. These results show that PAR1 activation plays a role in MT dynamics. Therefore, we suggest that PAR1 affects MT dynamics and therefore its modulation affects cell cycle and process formation.

In the structural aspect, we found a novel intracellular location of PAR1 in glioma cells especially during mitosis in close association with α-tubulin during the different stages of mitosis. MTs are responsible for several critical stages of cell division. They are involved in the assembly of the mitotic spindle, chromosome attachment and transport. The versatile function of the MTs during cell division is possibly due to tubulin post-translational modifications and the assistance of several MAPs [30]. Analysis of the PAR1 sequence using “microtubule-associated protein analyzer” [31] did not classify PAR1 as a MAP, raising an option of indirect PAR1 interaction with α-tubulin through binding to other MAPs. The membrane bound PAR1 is known to undergo differential intracellular trafficking and recycling processes. Upon activation, PAR1 is internalized to endosomes and is then degraded. Studies show that in some cases PARs can signal from the endosomes [32]. The intra-cellular location of PAR1 following activation is supported by previous findings, including its dimerization to protease-activated receptor 2 (PAR2), co-internalization and the recruitment of β-arrestins to the endosome [33]. Since β-arrestins are known to interact with the different elements of the cytoskeleton [34] our findings are in agreement with the literature and call for further characterization of PAR1 intracellular location. It is still an open question as to how specifically PAR1 interacts with MTs, whether via endosomal membrane during mitosis or via MAPs. Another question is the route by which PAR1 is transported to the MTs, whether it is synthesized and directed towards the MTs, or is redirected to the MTs following cell-surface recycling process. It was previously shown that PAR2 and other G-protein coupled receptors translocate on endosomes along MTs from the membrane to various sites inside the cell including the nucleus [35]. Analysis of the PAR1 sequence using the SBC NucPred tool [36] indicates potential NLS sequence (NucPred score of 0.15 which is above the threshold for identifying potential NLS), similar to PAR2. Therefore, it is possible that during mitosis PAR1 similarly translocate to the MTs. Further studies, including PAR1 expression downregulation and silencing, endosome characterization, and mapping of PAR1 binding proteins, are needed for in-depth understanding of PAR1/MT association.

The relevance of the PAR1/MT localization was evaluated by staining for several coagulation factors that are involved in PAR1 activation. We found factors from both the intrinsic and the extrinsic coagulation pathways on MT-associated structures in mitotic cells. Interestingly, in addition to thrombin, the main activator of PAR1, which was found to have a strong staining in the mitotic cells, its major nervous system-derived inhibitor, PN1, was also found in these experiments. This data suggests that PAR1 activation during mitosis is tightly regulated, and that additional coagulation factors and proteins may be involved in the process. One of these factors is aPC which binds to EPCR and alternatively activates PAR1 [26,37]. We found that both aPC and EPCR are present in the mitotic cells. Interestingly, aPC staining was closely located to the centrosome, resembling PAR1 staining. Therefore, we speculate that aPC is potentially modulate at least some of the steps of PAR1 activation during the cell cycle.

PAR1 is found to be a marker for invasiveness in many types of malignancies [16,17,[38], [39], [40]]. In addition, PAR1 also promotes malignant cell proliferation [16,17,41]. Similarly, MTs also play a role in cell migration, invasiveness, and proliferation [30,[42], [43], [44]]. Here, we demonstrate the association between PAR1 regulation and MTs function and provide a potential mechanistic explanation for the involvement of coagulation in GBM. Based on our research, we hypothesize that PAR1 is activated, possibly by thrombin or aPC, affects MAPs such as EB3, which leads to changes in MTs dynamics and therefore directly affects cell migration, proliferation, and invasiveness.

## 4 STAR methods

### 4.1 Resource availability

#### 4.1.1 Lead contact

Further information and requests for resources and reagents should be directed to and will be fulfilled by the lead contact, Dr. Shavit-Stein, Efrat.ShavitStein@sheba.health.gov.il.

## 5 Materials availability

This study did not generate new unique reagents.

## 6 Data and code availability

Accession numbers are listed in the key resources table. The DOI is listed in the key resources table. Microscopy data reported in this paper will be shared by the lead contact upon request.

DOIs are listed in the key resources table.

Any additional information required to reanalyze the data reported in this paper is available from the lead contact upon request.

No original code was generated in the present work.

## 7 Data

All data reported in this paper will be shared by the lead contact upon.

## 8 Code

This paper does not report original code.

## 9 Experimental model and subject details

### 9.1 Cells

Rat glioma C6 (CCL-107. ATCC) cells were purchased from ATCC company. The cells were evaluated for mycoplasma contamination, and genetic and phenotypic instability as accepted. The cells were grown in Dulbecco's modified Eagle's medium (DMEM; Bet Haemek, Biological Industries, Israel) supplemented with 10 % fetal bovine serum (Bet Haemek, Biological Industries, Israel), 1 % l-Glutamine (Bet Haemek, Biological Industries, Israel) and 0.1 % penicillin and streptomycin (Bet Haemek, Biological Industries, Israel). The cells were grown in a 37 °C and 5 % CO2-humidified atmosphere.

Mouse neuroblastoma N1E-115 cells (ATCC, Bethesda, MD; passage numbers from 10 to 13) were maintained in Dulbecco's modified Eagle's medium (DMEM), 10 % fetal bovine serum (FBS), 2 mM glutamine and 100 U/ml penicillin, 100 mg/ml streptomycin (Biological Industries, Beit Haemek, Israel). The cells were incubated in 95 % air/5 % CO2 in a humidified incubator at 37 °C. N1E-115 cells were plated on 35 mm dishes (81156, 60 μ-Dish, Ibidi, Martinsried, Germany) at a concentration of 254 cells/dish and then were differentiated with reduced FBS (2 %) and DMSO (1.25 %) containing medium during 5 days before transfection and 7 days before the experiment. On the day of the experiment differentiated N1E-115 cells were treated for 2 h with PAR1 antagonist diluted in DMSO (final concentration 100 nM and 1 μM) or DMSO alone (equally diluted).

### 9.2 Immunofluorescence

Cells were seeded in 24-well plates on glass coverslips (1 × 10^5^ cells/ml, 500 μl/well) covered with poly-l-lysine (Sigma, P4707) and allowed to attach for 24 h. The medium was then replaced with a serum-free medium. Following 24 h of incubation, cells were fixated using PFA 4 % (Electron Microscopy Sciences, 15710) and blocked for 1 h in PBS containing 10 % horse serum and 0.2 % Triton X-100 (Sigma, X-100). Cells were then incubated with primary antibodies (Table S1) at 4 °C overnight. On the following day, the cells were washed in PBS and incubated with the corresponding secondary antibody (DyLight 488/594 conjugated donkey anti-goat/rabbit/mouse IgG, Jackson ImmunoResearch, diluted 1:200 in PBS) for 1 h at room temperature. Then, the cells were washed, incubated with Hoechst (10 μg/ml, Sigma, 33258) for nuclear staining for 10 min, and washed again. Finally, the coverslips were dried and mounted with an anti-fading mounting medium (Fluoromount™, Sigma, F4680). Cells were imaged using a confocal microscope (Confocal microscope LEICA TCS SP5).

Co-localization analysis was performed utilizing the JACoP plugin in ImageJ software [45]. Results are presented as Pearson's correlation coefficient.

### 9.3 Cell viability assay

Cell viability was evaluated using CellTiter-Glo 2.0 Assay (Promega, #G5241). C6 cells were seeded (1 × 10^5^ cells/ml, 200μl/well) in a white 96-plate (Promega, E5650) and allowed to attach for 24 h. The medium was then replaced by serum-free DMEM (100 μl) containing treatments (PAR1 antagonist SCH79797 (Tocris) [100 nM], or thrombin (Sigma, T4648) [1 U/ml]). Following 24 h, 100 μL of CellTiter-Glo 2.0 reagent was added to each well and the microplate was placed on a plate shaker for 2 min and incubated at room temperature for 10 min. Luminescence was then detected on the SpectraMax iD5 reader (Molecular Devices).

### 9.4 Thrombin activity

Thrombin activity was measured using a fluorometric assay based on the cleavage rate of the synthetic substrate Boc-Asp (OBzl)-ProArg-AMC (I-1560; Bachem, Bubendorf, Switzerland) as described previously [46]. C6 cells were seeded at different densities (3 × 10^3^, 6 × 10^3^, 1.2 × 10^4^, 2.5 × 10^4^, 5 × 10^4^, 1 × 10^5^ cells/ml, 200 μl/well) and allowed to attach for 24 h. The medium was then replaced by serum-free DMEM (100 μl) and 24 h later, the medium was transferred to a black 96-well microplate. The substrate (14 μM) was added to each well and fluorescence was measured using a microplate reader (Tecan; infinite 200; Switzerland) with excitation and emission filters of 360 ± 35 and 460 ± 35 nm, respectively. Known concentrations of bovine thrombin (Sigma, T4648) were used for calibration.

### 9.5 Scratch assay

Cells were seeded in 24-well plates at a density of 5 × 10^4^ cells/well and allowed to attach for 24 h. Cells were cultures in an FCS-free medium for 24 h. A scratch was established by scraping using 10 μl pipette tip. Cells were washed twice with warm PBS and fresh FCS-free medium containing treatment (PAR1 antagonist SCH79797 (Tocris) [100 nM], thrombin (Sigma, T4648) [1U/ml]) was added. Image acquisition of wound gap closure (%) was carried out following 4 h of incubation using a Nikon Eclipse microscope. Images were analyzed using ImageJ software [47].

### 9.6 Fluorescence-activated cell sorting (FACS)

Cells were seeded in 6-well plates at a density of 1 × 10^5^ cells/well and allowed to attach for 24 h. The medium was then replaced by serum-free DMEM (500 μl) containing treatment (PAR1 antagonist SCH79797 (Tocris) [100 nM], thrombin (Sigma, T4648) [1U/ml]) for 24 h. Cells were collected and fixed using 70 % ethanol at 4 °C overnight. Following fixation, cells were washed with PBS and stained with Tali™ (Thermo Fisher Scientific, A10798) reagent according to manufacturer instructions.

### 9.7 Real-time polymerase chain reaction (RT-PCR)

Cells were seeded in 6-well plates at a density of 1 × 10^5^ cells/well and allowed to attach for 24 h. The medium was then replaced by serum-free DMEM (3 ml) containing treatment (PAR1 antagonist SCH79797 (Tocris) [100 nM], thrombin (Sigma, T4648) [1U/ml]) for 3 h. Following the treatment, the cells were collected, and the RNA was extracted and cleaned according to the Bio-Rad Aurum 732–6820 kit instructions (Bio-Rad Laboratories, Hercules, CA, USA). Two micrograms of total RNA were used for reverse transcription using high-capacity cDNA reverse transcription kit (Applied Biosystems). Quantitative real-time polymerase chain reaction (RT-PCR) was performed on the StepOne™ Real-Time PCR System (Applied Biosystems, Rhenium, Israel) using Fast SYBR Green Master (ROX) (Applied Biosystems). Hypoxanthine guanine phosphoribosyltransferase (HPRT) served as a reference gene in this analysis. A standard amplification program was used (1 cycle of 95 °C for 20 s (s) and 40 cycles of 95 °C for 3 s and 60 °C for 30 s). The primers used in this analysis are listed in Table S2. The results were normalized to reference gene expression within the same cDNA sample and calculated using the ΔCt method with results reported as fold changes relative to control samples.

### 9.8 Live imaging of N1E-115 cells

N1E-115 cells were transfected (jetPEI; Polyplus Transfection, Illkirch, France) with a 1 μg pTagRFP-EB3 plasmid (FP365, Evrogen, Moscow, Russia). 48 h after transfection, cultured N1E-115 cells were incubated at 37 °C with a 5 % CO2/95 % air mixture in a thermostatic chamber placed on the stage of a Leica TCS SP5 confocal microscope [objective 100× (PL Apo) oil immersion, NA 1.4]. Time-lapse images were automatically captured every 3 s for 1 min using the Leica LAS AF software. Data was collected and independently analyzed by Imaris software.

### 9.9 Quantification and statistical analysis

Statistical analyses and graphs were conducted using GraphPad Prism (version 7.00 for Windows, GraphPad Software, La Jolla California USA, www.graphpad.com). Paired t-tests, one-way ANOVA, and two-way ANOVA followed by a post hoc test were applied to normally distributed data sets. One-way ANOVA was followed by either Dunnett's or Tukey's post hoc analyses. Two-way ANOVA was followed by Sidak's post hoc analysis. Results of post-analyses are presented only when ANOVA results show statistical significance. The Mann-Whitney test was applied to non-normal distributed data sets. Results are expressed as mean ± SEM, p values < 0.05 were considered significant. The numbers of subjects on which analysis was conducted are reported in the figure legend.

## Key resource table

**Table:** 

REAGENT or RESOURCE	SOURCE	IDENTIFIER
Antibodies		
Rabbit anti PAR1	MyBiosource, MBS9201361	–
Rabbit anti PAR1	MyBiosource, MBS273633	–
Mouse anti α-tubulin	Santa cruz, sc-5286	AB_628411
Goat anti Thrombin	Santa cruz, sc-23355	AB_671340
Goat anti PN1	Santa cruz, sc-32454	AB_2187059
Goat anti TF	Santa cruz, sc-23596	AB_2209608
Rabbit anti FVIII	Novus, NB100-91761	AB_1216686
Rabbit anti aPC	ABBiotec, 251142	AB_10636205
Goat anti EPCR	Santa cruz, sc-23575	AB_2172562
Chemicals, peptides, and recombinant proteins		
PAR1 antagonist SCH79797	Tocris	
Thrombin, bovine	Sigma, T4648	
Boc-Asp (OBzl)-ProArg-AMC	I-1560; Bachem, Bubendorf, Switzerland	
Bio-Rad Aurum 732–6820 kit instructions	Bio-Rad Laboratories, Hercules, CA, USA	
StepOne™ Real-Time PCR System	Applied Biosystems, Rhenium, Israel	
Critical commercial assays		
CellTiter-Glo 2.0 Assay	Promega, #G5241	
Experimental models: Cell lines		
Rat glioma C6	ATCC company, CCL-107	CVCL_0194
Software and algorithms		
JACoP plugin in ImageJ software [45]		
ImageJ software		SCR_003070
GraphPad Prism	version 7.00 for Windows, GraphPad Software, La Jolla California USA, www.graphpad.com	SCR_002798

## CRediT authorship contribution statement

**Valery Golderman:** Writing – original draft, Visualization, Methodology, Investigation, Formal analysis, Data curation, Conceptualization. **Shany Guly Gofrit:** Writing – review & editing, Writing – original draft, Formal analysis, Conceptualization. **Yanina Ivashko-Pachima:** Visualization, Investigation, Formal analysis. **Illana Gozes:** Writing – review & editing, Conceptualization. **Joab Chapman:** Writing – review & editing, Methodology, Conceptualization. **Efrat Shavit-Stein:** Writing – review & editing, Methodology, Formal analysis, Conceptualization.

## Declaration of competing interest

The authors declare the following financial interests/personal relationships which may be considered as potential competing interests: Efrat Shavit-Stein reports financial support was provided by Israel Ministry of Innovation Science & Technology. Efrat Shavit-Stein, Joab Chapman has patent #PAR1 modulating molecules: “Compositions and methods for treating glioblastoma” issued to US-2021228681-A1.
