# Supplementary material for: The thrombin receptor (PAR1) is associated with microtubules, mitosis and process formation in glioma cells
Source: Heliyon. 2024 Jun 19;10(12):e33329. doi: 10.1016/j.heliyon.2024.e33329 (PMC11254606; doi:10.1016/j.heliyon.2024.e33329)
Supplement: Multimedia component 1 [file mmc1.docx]

**Tables**

Table S1

| Antibody | Dilution | Company (# catalog) | Comments |
| --- | --- | --- | --- |
| Rabbit anti PAR1 | 1:100 | MyBioSource, MBS9201361 | Immunogen AA Sequence positions: 10-39 |
| Rabbit anti PAR1 | 1:500 | MyBioSource,  MBS273633 | Immunogen AA Sequence positions: 1-90 |
| Mouse anti α-tubulin | 1:400 | Santa Cruz Biotechnology, sc-5286 |  |
| Goat anti Thrombin | 1:100 | Santa Cruz Biotechnology, sc-23355 |  |
| Goat anti PN1 | 1:50 | Santa Cruz Biotechnology, sc-32454 |  |
| Goat anti TF | 1:100 | Santa Cruz Biotechnology, sc-23596 |  |
| Rabbit anti FVIII | 1:100 | Novus, NB100-91761 |  |
| Rabbit anti aPC | 1:200 | ABBiotec, 251142 |  |
| Goat anti EPCR | 1:100 | Santa Cruz Biotechnology, sc-23575 |  |
| Mouse anti HA | 1:100 | BioLegend, 901501 |  |

Table S1: Primary antibodies’ list.

Table S2

| Gene | Forward | Reverse |
| --- | --- | --- |
| HPRT | GATTAGCGATGATGAACCAGGTT | CCTCCCATCTCCTTCATGACA |
| PAR1 | GCCTCCATCATGCTCATGAC | AAAGCAGACGATGAAGATGCA |
| FX | GTGGCCGGGAATGCAA | AACCCTTCATTGTCTTCGTTAATGA |
| EB1 | GCAGTGGCTCCTTCTCTTGT | TCTGTGTTGCAATGGGTCTC |
| EB3 | AAAGGATTACAACCCTCTGCTG | ACGTCCTCTGTGGAACTG |

Table S2: Primers sequence list.

**Figures**

**
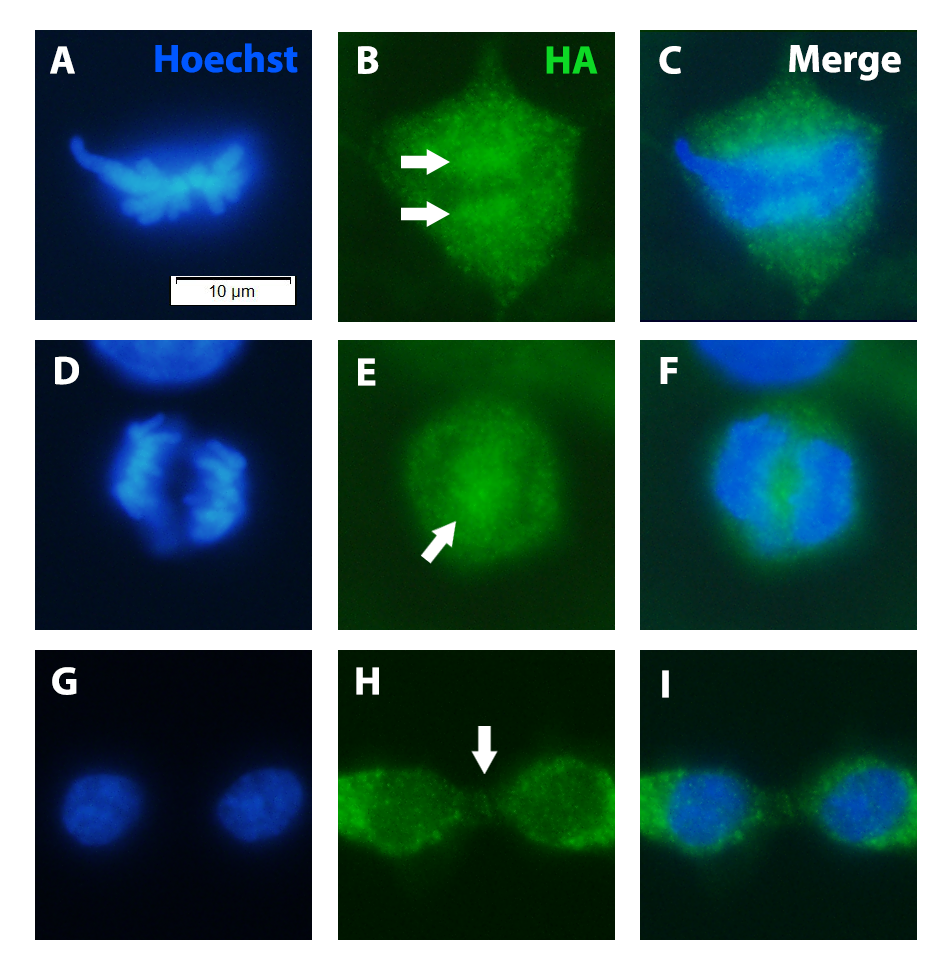
**

**Fig. S1: HA localization in transfected C6 cells:** Representative images of transfected C6 cells stained to Hoechst (blue) and HA (green). A-C. Localization of HA in metaphase – HA staining localized close to the metaphase plate, as indicated by the white arrows, similar to PAR1 staining. D-F. Localization of HA in anaphase – HA staining localized to the polar MTs, as indicated by the white arrow, similar to PAR1 staining. G-H. Localization of HA in telophase – HA staining localized close to PAR1 staining with weak staining at the central spindle, as indicated by the white arrow. Scale bar – 10 µm.
